# Eating Behavior (Duration, Content, and Timing) Among Workers Living under Different Levels of Urbanization

**DOI:** 10.3390/nu12020375

**Published:** 2020-01-31

**Authors:** Natalia M. Tiuganji, Patricia Nehme, Elaine C. Marqueze, Cheryl M. Isherwood, Andressa J. Martins, Suleima Vasconcelos, José Cipolla-Neto, Arne Lowden, Debra J. Skene, Claudia R. C. Moreno

**Affiliations:** 1 School of Public Health, University of São Paulo, 715 Av. Dr. Arnaldo, São Paulo SP 01246-904, Brazil; nataliamitie@hotmail.com (N.M.T.); patricianehme@usp.br (P.N.); ecmarqueze@gmail.com (E.C.M.); andressajuliane@gmail.com (A.J.M.); 2 Epidemiology, Public Health Graduate Program, Catholic University of Santos, 300 Av. Conselheiro Nébias, Santos SP 11045-003, Brazil; 3 Faculty of Health and Medical Sciences, University of Surrey, Guildford, Surrey GU2 7XH, UKd.skene@surrey.ac.uk (D.J.S.); 4 Department of Health Science and Sports Center, Federal University of Acre, Rodovia BR 364, Km 04–Rio Branco–AC 69920-900, Brazil; suleimav@hotmail.com; 5 Institute of Biomedical Sciences, University of São Paulo, 1524 Av. Prof. Lineu Prestes, São Paulo SP 05508-000, Brazil; cipolla@icb.usp.br; 6 Stress Research Institute, Department of Psychology, University of Stockholm, SE-106 91 Stockholm, Sweden; arne.lowden@su.se

**Keywords:** meal timing, eating duration, urbanization, food content

## Abstract

Urbanization has contributed to extended wakefulness, which may in turn be associated with eating over a longer period. Here, we present a field study conducted in four groups with different work hours and places of living in order to investigate eating behavior (duration, content, and timing). Anthropometric measures were taken from the participants (rural (*n* = 22); town (*n* = 19); city-day workers (*n* = 11); city-night workers (*n* = 14)). In addition, a sociodemographic questionnaire was self-answered and 24-h food recalls were applied for three days. The 24-h food recalls revealed that fat intake varied according to the groups, with the highest consumption by the city-day workers. By contrast, city-day workers had the lowest intake of carbohydrate, whereas the rural group had the highest. In general, all groups had some degree of inadequacy in food consumption. Eating duration was negatively correlated with total energy intake, fat, and protein consumption in the rural and town groups. There was a positive correlation between body mass index and eating duration in both city groups. The rural group had the earliest start time of eating, and this was associated with a lower body mass index. This study suggested that food content and timing, as well as eating duration, differed according to place of living, which in turn may be linked to lifestyle.

## 1. Introduction

The urbanization process, as well as the use of electricity and the technologies derived from this, allow extended wakefulness and provide the opportunity to carry out activities after sunset [1,2,3]. Studies conducted in communities with and without access to electricity have shown that electric light can lead to delayed sleep onset and, consequently, to reduced sleep duration [4,5,6]. Moreno et al. [5] also observed that rubber tappers from the Amazon forest with access to electricity had delayed melatonin timing compared to the non-exposed group. In addition, there is evidence that urbanization and access to electricity may be associated with increased body weight and related comorbidities due to desynchronization of circadian rhythms [7,8] and insufficient sleep duration [9,10,11,12].

The increase in comorbidities due to prolonged wakefulness may be exacerbated by the extension of feeding at inappropriate times for the physiological and metabolic processes regulated by the circadian timing system [13]. This scenario appears to be worse when associated with shift work [14,15]. Recent evidence demonstrates that eating close to or after melatonin onset, i.e., the time when the melatonin concentration is rising, may affect the thermic effect of food [16]. In addition, body mass index (BMI) was associated with mealtimes, whereby later mealtimes were associated with increased body fat [16]. Another study exposed two groups of obese women to similar hypocaloric diets that differed in the distribution of calories throughout the day (BF group: Higher calories at breakfast; group D: Higher calories at dinner). Greater weight loss was observed in the BF group, as well as higher reductions in fasting serum glucose, insulin, total cholesterol, and the homeostatic model assessment of insulin resistance (HOMA-IR), and a slight increase in high density lipoprotein cholesterol (HDL) compared to the D group [17]. Thus, feeding during the biological night, characterized by melatonin synthesis, may be a risk factor for cardiovascular problems [15,17], overweight [15,16,17], and other comorbidities [15,18]. This might be related to less time for anabolic pathways, which may mean that the repair processes are sacrificed as well as a suboptimal metabolic state for food consumption [15,18,19,20,21].

In parallel with mistimed feeding, the duration of food intake is longer, allowing for greater energy consumption, which, per se, correlates with negative health outcomes, even when total energy consumption remains constant [22,23]. Thus, while on the one hand there is evidence that prolonged wakefulness is associated with urbanization and electrification [5], on the other hand, it has been shown that longer wakefulness can alter dietary patterns [24]. Although studies comparing food consumption in urban and rural areas are scarce, in Tanzania, Njelekela et al. [25] reported a significant association between urban living and higher socioeconomic level with more meat and beverages in the diet. The authors also found lower consumption of traditional foods and less basic food preparations to be associated with urban living and higher socioeconomic status.

Thus, the main hypothesis of the present study was that residents in locations with a high level of urbanization are more likely to have poor eating habits. Urban dwellers would therefore be expected to have higher levels of overweight or obesity compared to individuals living in rural environments. Longer eating durations and night work may also be associated with poor eating habits and overweight.

## 2. Methods

### 2.1. Population

This cross-sectional design study involved 68 workers living in two different states of Brazil (Acre and São Paulo), thereby encompassing different levels of urbanization. The statistical power of the sample was 94.9%, calculated a posteriori with an alpha error of 5%, considering the excessive consumption of fat (mean = 51.7%) as a proxy of poor food habits.

The participants were allocated to four groups: Rural (RAC; *n* = 22) and small town dwellers (TAC; *n* = 19) from Acre (AC), and city dwellers engaged in daytime work (DSP; *n* = 11) and night work (NSP; *n* = 14) from São Paulo (SP) city. All participants had access to electricity in their homes. Both groups from Acre were residents of the town of Xapuri (latitude: 10°39’06″ S), in the northern region of Brazil, which has 16,091 inhabitants, comprising 5761 from the rural area and 10,330 (64%) from the town area [26]. RAC participants worked during the day on subsistence agriculture and extractivist activities (gathering nuts) and lived in the forested region. TAC participants lived in the urban area of the town and worked during the day as teachers at a public elementary school.

The DSP and NSP groups worked at a public hospital as nurses, technicians, nursing assistants, or laboratory technicians. São Paulo (latitude: 23°54’89″ S) is the capital of São Paulo state, and has 12,106,920 inhabitants in the urban area. The DSP schedule was 12 h of work (07:00–19:00 h) followed by 36 h off, and night workers (NSP) followed the same schedule (18:00–06:00 h or 19:00–07:00 h).

### 2.2. Ethical Aspects

The study was approved by the Research Ethics Committee of the School of Public Health, University of São Paulo, Brazil (process numbers 1.581.480 and 1.127.697) and by the Ethics Committee of the Hospital (process number 463922147/001000) in accordance with the ethical standards laid down in the 1964 Declaration of Helsinki and its later amendments. All participants provided written informed consent.

### 2.3. Data Collection

Anthropometry measurements were taken from all participants and BMI was used as a proxy measure for metabolic health. BMI was categorized according to the classification criteria of the World Health Organization (WHO) [27], namely: Low weight (BMI < 18.5 kg/m^2^), healthy weight (18.5 < BMI < 24.9 kg/m^2^), overweight (25.0 ≤ BMI < 29.9 kg/m^2^), and obesity (BMI ≥ 30 kg/m^2^).

Food and beverage consumption were evaluated based on three 24-h food recall interviews applied on non-consecutive days, including 1 day off and 2 work days. This method allowed the estimation of habitual food consumption. It included questions related to how meals were prepared and served, the place of consumption, and amounts consumed in household measures from wake-up time to bedtime. All the 24-h recalls were conducted by trained nutritionists and household measures subsequently converted into international standard measures [28,29,30].

Nutritional composition analysis was performed using the Nutrition Data System Research version 2016 (NDSR) [31] software, Nutrition Coordinating Center, University of Minnesota, Minneapolis, MN, USA. The composition and preparation methods of typical Brazilian foods were added to the database, using food labels and the Brazilian National Table of Food Content (TACO) (2011) [32].

Eating duration was calculated as the period from the first caloric intake after wake-up to the last caloric intake before sleep onset. Eating duration was determined for each day and then averaged (two working days and one day off) for each person. Averages from each group were then used for the analysis of eating duration. Regarding the timing of meals, two different analyses were performed. Firstly, the average consumption (content and timing) for the three recorded days was calculated for each group and the start and end times (and mid-point) of eating were calculated [33]. Secondly, meal timing was broken down into four 6-h periods throughout the day (00:01–06:00 h; 06:01–12:00 h; 12:01–18:00 h and 18:01–00:00 h) to determine the number of meals and percentage of caloric intake per period. This calculation was first done for each recorded day per person, and the average for three days was then calculated. The procedure to break down the meal timing into periods was used previously [34,35]. In addition, the specific characteristics of the study groups were taken into account as the start and end times of eating, and the intervals without food intake differed.

Data were collected in Acre in both 2015 (September and October) and 2016 (July and August). The average time of sunrise was 05:19 h (±10 min) and sunset was 17:29 h (±0.5 min) in September/October, whereas in July/August, sunrise was at 05:49 h (±5 min) and sunset at 17:27 h (±3 min). In São Paulo, data were collected between August 2016 and April 2017. Sunrise ranged from 05:34 h to 06:57 h and sunset ranged from 17:39 h to 19:57 h [36].

### 2.4. Statistical Analysis

Descriptive analysis of the data was initially performed that included calculating the percentage intake of each macronutrient (protein, fat, and carbohydrate) per group and location. Subsequently, these percentages were compared to dietary reference intakes (DRIs) [37], using the acceptable macronutrient distribution range (AMDRs) for adults, regardless of sex. The AMDR for fat and carbohydrate was estimated to be 20%–35% and 45%–65% energy for adults, respectively. Protein accounted for 10%–35% energy in adults.

In order to investigate the differences in consumption among the groups according to the AMRDs and level of significance of these differences, a general linear model was performed (GLM –post-hoc LSD) using age as a covariate. Fisher’s exact test was also performed to compare the proportions of macronutrients according to the groups. Given the data distribution of the eating duration and macronutrient variables, possible correlations between these were verified (Pearson or Spearman’s correlation coefficient tests were performed according to the normal distribution of data). The ANOVA or Kruskal–Wallis (KW) tests were performed to evaluate the mean difference in eating episodes and calorie intake for different time periods between urban and rural workers in Acre and São Paulo. All analyses were performed using Statistica, TIBCO Software Inc., Palo Alto, CA, USA (version 12) and Stata, Stata Corp., College Station, TX, USA (version 12.0) software.

## 3. Results

### 3.1. Sociodemographic and Anthropometric Characteristics

The groups from Acre (rural-RAC; *n* = 22, and town dwellers-TAC; *n* = 19) were male and the mean age was 41.6±10.9 (mean ± SD) and 38.8 ± 8.2 (mean ± SD) years for the RAC and TAC groups, respectively. In São Paulo, the groups were female and the mean age was 41.6 ± 6.1 (mean ± SD) years for city dwellers working during the daytime (DSP) and 39.1 ± 5.2 (mean ± SD) years for those working at night (NSP). Other sociodemographic characteristics are shown in Table 1.

**Figure 1:**
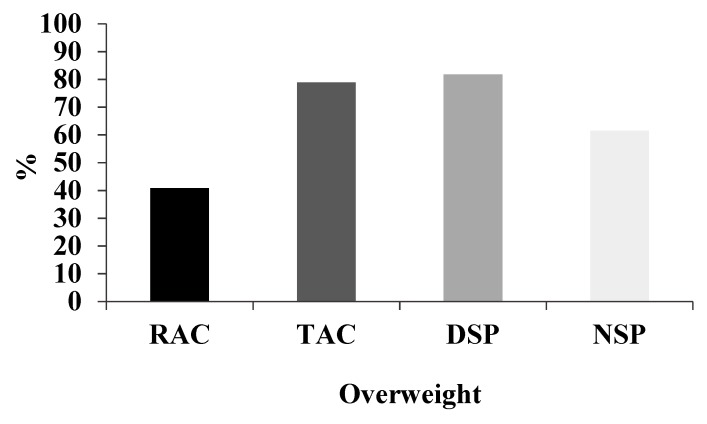
Percentage overweight among urban and rural workers in Acre (AC) and São Paulo (SP). Rural = RAC, town = TAC, city-day workers = DSP and city-night workers = NSP. Overweight = BMI > 30 kg/m^2^. Fisher’s exact test revealed differences between groups, *p* = 0.04.

The mean (SD) BMI was 24.95 kg/m^2^ (3.27 kg/m^2^) for RAC, 28.07 kg/m^2^ (4.22 kg/m^2^) for TAC, 30.6 kg/m^2^ (7.36 kg/m^2^) for DSP, and 26.55 kg/m^2^ (3 kg/m^2^) for the NSP group. The urban groups (DSP, TAC, NSP) had the highest proportion of overweight participants (BMI > 25kg/m^2^), with an 82% rate in the DSP, 79% in the TAC, and 62% in the NSP, whereas only 41% of the rural group (RAC) were overweight (Figure 1).

### 3.2. Energy and Macronutrient Consumption

**Figure 2:**
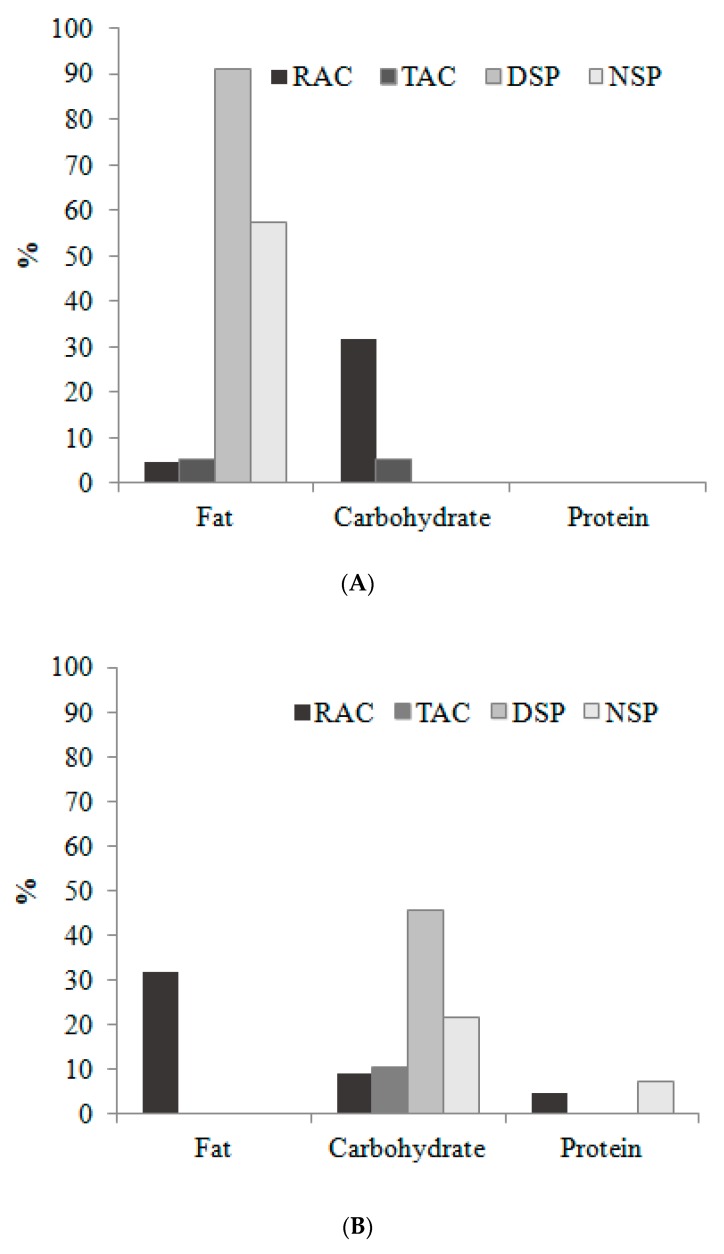
Macronutrient consumption among rural and urban workers in Acre (AC) and São Paulo (SP). (**Panel A**) Prevalence of excessive consumption; (**Panel B**) Prevalence of insufficient consumption. Rural = RAC, town = TAC, city-day workers = DSP and city-night workers = NSP. RAC = 22; TAC = 19; DSP = 11; and NSP = 14 workers. The acceptable macronutrient distribution range (AMDR)% energy estimates for adults; carbohydrate (45%–65%), fat (20%–35%) and protein (10%–35%). No values mean no consumption.

Group comparisons using a proportion test revealed a higher proportion of fat consumption among city dwellers (DSP and NSP) (Figure 2A), whereas the rural group (RAC) had insufficient intake of fat (Figure 2B) (Fisher’s exact test *p* < 0.001). On the other hand, carbohydrate consumption was excessive for 32% of the RAC group (Figure 2A) and insufficient for 45% of the DSP group (Fisher’s exact test *p* = 0.011) (Figure 2B).

**Figure 3:**
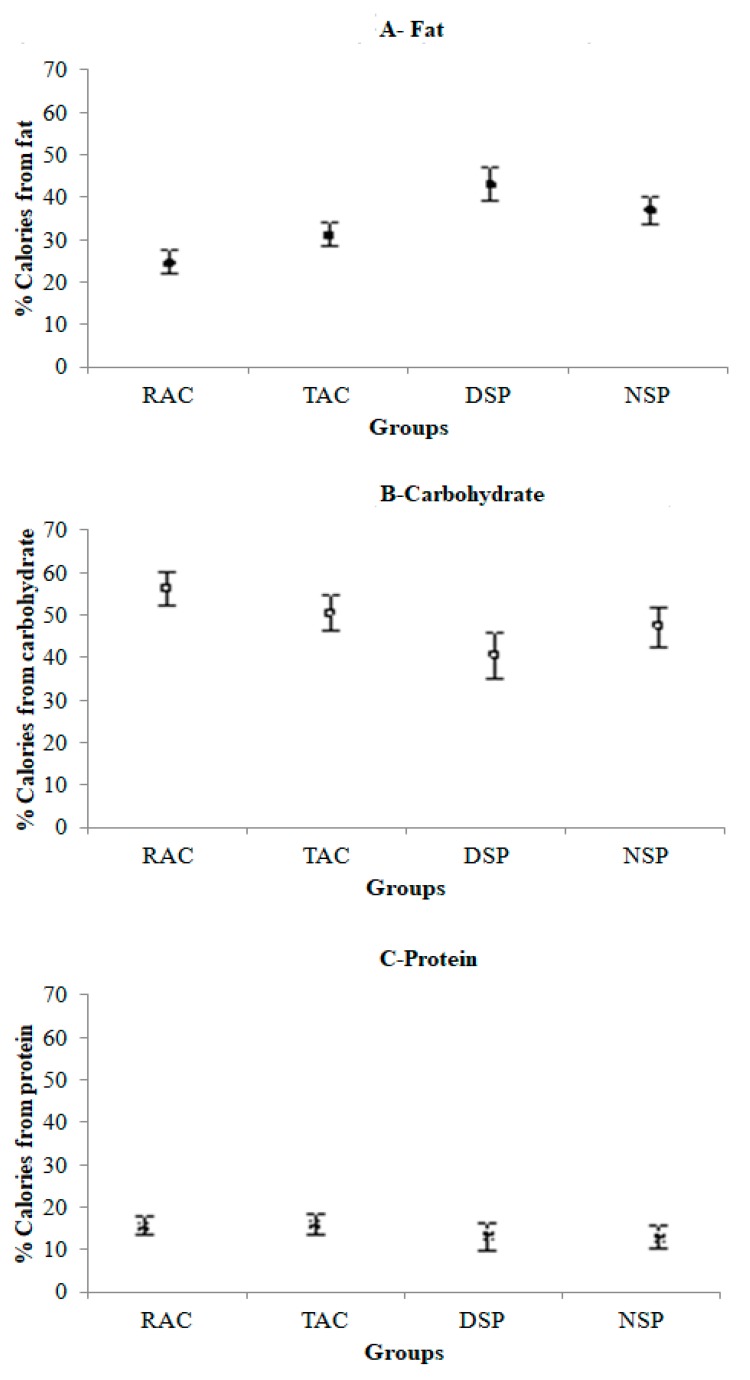
Calories derived from each macronutrient, according to group and adjusted for age. (**A**) Fat (GLM: F(3, 61) = 23.12, *p* < 0.001); (**B**) Carbohydrate (GLM: F(3, 61) = 8.35, *p* < 0.001); (**C**) Protein (GLM: F (3, 61) = 1.50, *p* = 0.225). RAC, *n* = 22; TAC, *n* = 19; DSP, *n* = 11; NSP, *n* = 14. Rural = RAC, town = TAC, city-day workers = DSP and city-night workers = NSP.

The percentage of energy derived from each macronutrient varied according to the groups studied. An LSD post-hoc test showed that fat consumption differed significantly between the groups (Figure 3A). Moreover, the rural group (RAC) had the highest carbohydrate consumption (Figure 3B). No differences among the groups were observed regarding protein consumption (Figure 3C).

### 3.3. Eating Duration and Timing

**Figure 4:**
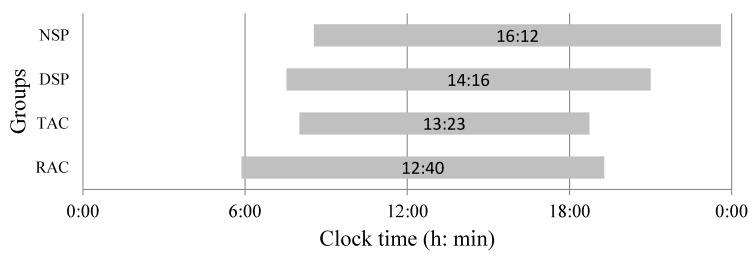
Average eating timing (h:min) and duration (h), according to groups. Mid-points (h:min) of the eating period are shown within the bars. Rural = RAC, town = TAC, city-day workers = DSP and city-night workers = NSP. Mean ± SD eating duration: NSP: 14.6±1.4 h; DSP: 13.2±0.6 h; TAC: 11.7±0.25 h; RAC: 13.4±0.3 h.

As expected, the NSP group consumed food over the longest period of time per 24 h (14.6 ± 1.4 h; mean ± SD)), ending later than the other groups (on average, started at 08:33±1:47 h (mean ± SD) and ended at 23:10 ± 3:09 h (mean ± SD)). The RAC group began eating much earlier (on average, started at 05:52 ± 0:40 h (mean ± SD) and ended at 19:17 ± 0:57 h (mean ± SD)) than the other groups (Figure 4). Although the variability of the duration of food intake of the Acre groups was lower than the São Paulo groups, this variability does not interfere with macronutrient consumption, since carbohydrate consumption was higher in the Acre groups (Figure 3B). In addition, fat consumption in the TAC and NSP groups was very similar (Figure 3A).

Pearson’s correlation analysis showed that the duration of the eating period was negatively correlated with the total energy intake, and fat and protein consumption in the RAC and TAC groups (total calories: *r* = −0.44, *p* = 0.004; fat: *r* = −0.46, *p* = 0.003; protein: *r* = −0.33, *p* = 0.03). Carbohydrate consumption exhibited a similar trend as the other macronutrients, measured by Spearman’s coefficient (*rho* = −0.30, *p* = 0.06). There was no correlation, however, between the eating duration and BMI for the RAC and TAC groups.

There was no correlation between the eating duration and protein or fat consumption in the DSP and NSP groups. However, Spearman’s correlation showed a positive association between eating duration and both BMI (*rho* = 0.48, *p* < 0.05) and carbohydrate consumption (*rho* = 0.57, *p* = 0.003) in the DSP and NSP groups.

**Figure 5:**
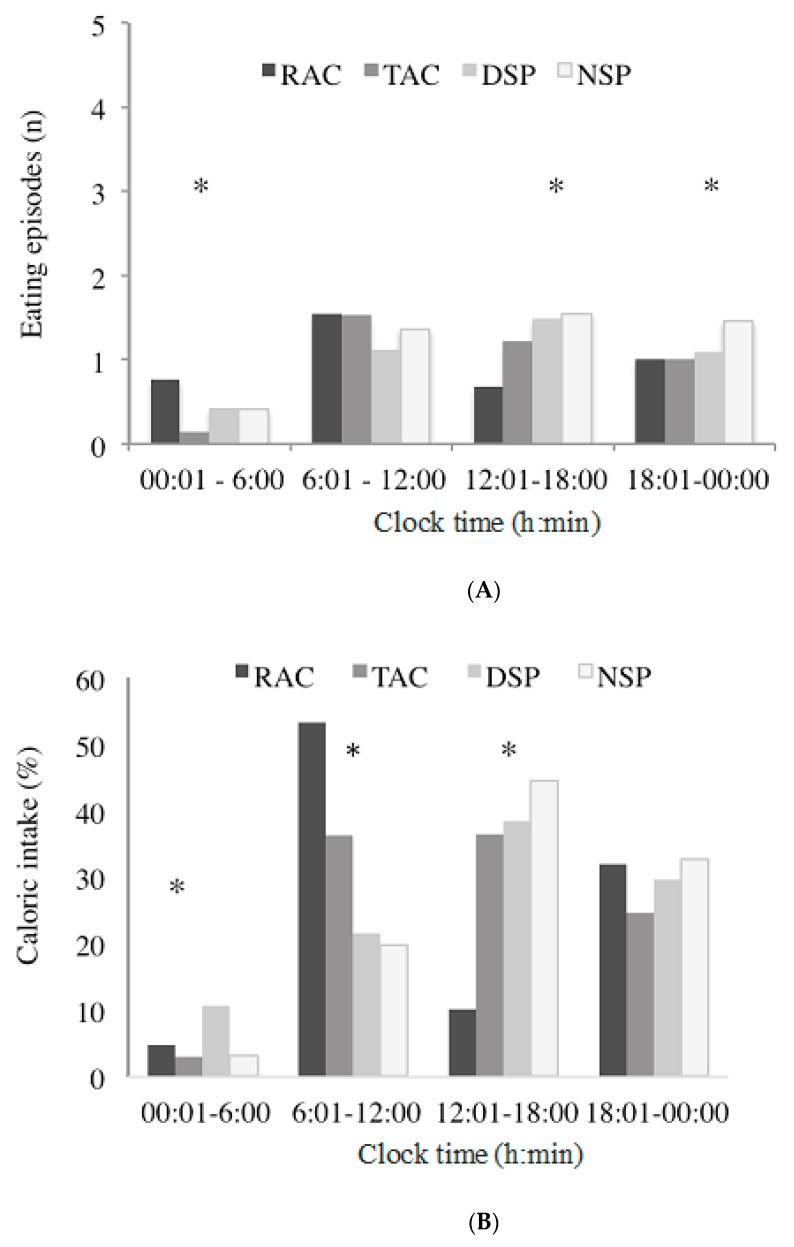
Number of eating episodes and calorie intake during different time periods among urban and rural workers in Acre and São Paulo. (**A**) number of eating episodes per day; (**B**) caloric intake (%) according to time of day. Eating episodes: 00:01–06:00 h (*KW *p* < 0.01); 12:01–18:00 h (*ANOVA *p* < 0.01); 18:01–00:00 h (*KW *p* < 0.01). Calorie intake: 00:01–06:00 h (*KW *p* < 0.01); 06:01–12:00 h (*ANOVA *p* < 0.01); 12:01–18:00 h (*KW *p* < 0.01).

Meal timing was broken down into four separate 6-h periods throughout the day (00:01–06:00 h; 06:01–12:00 h; 12:01–18:00 h; and 18:01–00:00 h) and calorie intake measured for each period, according to the groups (Figure 5). All groups had a similar number of meals or eating episodes in the morning (06:01–12:00 h); however, the RAC group had more eating episodes (on average) before 06:00 h (*p* < 0.01) (Figure 5A). Calorie intake varied among the groups for the different time periods, except from 18:01 to 00:00 h, for which there were no differences among the groups (Figure 5B). The RAC group had the highest calorie intake in the morning (06:01–12:00 h) (Figure 5B).

## 4. Discussion

The large variety of Brazilian communities provides an opportunity to assess the effect of urbanization on eating behavior. In the present study, we assessed eating duration together with meal content and timing in people living in places with different levels of urbanization. Analysis of the macronutrient consumption among rural and urban dwellers, living in different locations in Brazil (Acre and São Paulo), revealed that all groups had some degree of inadequacy in food consumption. When compared to dietary reference intake (DRI) [37] values, fat inadequacy was found to be more exacerbated among city workers in São Paulo (DSP and NSP), particularly in the DSP group.

Regarding eating duration, we observed that, in the case of Acre dwellers (RAC and TAC), the longer the eating duration, the lower the calorie intake, as well as fat and protein intake. Surprisingly, there was no correlation between eating duration and BMI for the RAC and TAC groups. This result may represent a peculiarity of this population, which eats the first meal of the day very early in the morning (RAC) and/or eats the last meal early in the evening (TAC). By contrast, for São Paulo city workers (DSP and NSP), there was the expected positive correlation between eating duration and carbohydrate consumption with BMI. It has been postulated that eating late meals may represent a risk factor for metabolic disorders [38], although the underlying mechanism has not yet been fully elucidated. At night, the energy expenditure in response to meals is reduced [16]. Thus, when food is consumed during the night, this may have an impact on the diversity and amount of salivary microbiota (among other possible effects), which may in turn have negative effects on human metabolism [39]. Moreover, the composition of the diet represents a fundamental factor for the occurrence of negatives changes in the intestinal microbiota. The intake of a high-fat or high-sugar diet, associated with exposure to a light/dark cycle inversion, is capable of generating significant changes in the intestinal microbiota due to an increase in the number of bacteria associated with inflammation, according to the study of Voigt et al. (2014) [40].

Recent studies have shown that there is a strong relationship between energy regulation and the circadian clock at all levels (molecular, physiological, and behavioral), suggesting that the timing of feeding per se plays a significant role in weight gain [41,42]. Moreover, satiety hormones have time-of-day variations that may contribute to the observed weight gain [43]. In view of this, we expected to find a positive correlation between eating duration and energy intake and macronutrient consumption. It is possible that the very early morning eating period in the RAC group contributes to the lower food consumption observed. This could mean that timing of eating, especially early eating, is even more important than the eating duration period. In a recent review of experimental animal models that mimic the conditions of shift work, it was suggested that shifting the time of food intake might be a determining factor for the loss of internal synchrony. This effect could be related to a differential response of individual organs to entraining food signals [44], as recently demonstrated in humans experiencing a 5-h delay in mealtimes [45]. Nevertheless, it should be mentioned that differences in the socioeconomic conditions among the groups might also contribute to the observed difference in fat consumption [46]. Moreover, low fat consumption, as well as physical effort at work, may contribute to the lower percentage of overweight and obesity among this rural population relative to the other groups [46,47]. Although the differences in physical activity might be seen as a limitation of this study, its main focus was to analyze the choice of foods, food content, and feeding times, therefore it seems unlikely that physical activity may have some influence on these factors. Another issue is sex differences in food intake. It has been reported that women are more likely to exceed recommendations for fat consumption. Although, in this study, we used food intake recommendations regardless of sex, these differences may be a limitation of this study.

It seems that the RAC group had a strategy of eating more calories in the morning than the other groups due to the physical work tasks ahead. By contrast, the TAC was the second most overweight group. Different lifestyles between the groups might be a possible explanation for this difference in overweight. Among city workers in São Paulo (DSP and NSP), we observed a high prevalence of overweight, regardless of what shift they worked. There was an expected positive correlation between BMI and eating duration for both groups of workers from São Paulo. We have previously shown an association between town residents from Acre and the presence of risk factors for metabolic disorders, such as overweight [48]. These findings are corroborated by studies that show that a nutritional transition is followed by weight gain of the population [49]. More than 80% of the DSP group were overweight, although the percentage of overweight among the NSP group was also high.

A consensus of the Working Time Society states that there is strong evidence linking shift work (including night work) and metabolic disorders (type 2 diabetes; metabolic syndrome) but weak evidence regarding obesity [50]. Although some studies have suggested that the night shift is potentially associated with the presence of obesity [51,52], night shifts can be part of a rotating schedule that includes day shifts, which may also play a role in the development of obesity [51]. Our sample of city daytime workers had a high level of overweight, similar to the rate reported by Chin et al. [52], who analyzed several occupational factors associated with obesity and also physical activity during leisure time. Current evidence from this study suggests that daytime workers are more likely to engage in physical activity. However, the responses regarding occupational aspects seem to be affected by the holding of management/supervision positions and working long hours (more than 40 h per week). These data provide strong evidence that obesity is a multifactorial disease influenced by several aspects of contemporary society, such as diet, sleep behavior, occupational aspects, and others. On the other hand, Sun et al. [53] found a positive correlation between night work and the development of overweight and obesity among 3871 Chinese industrial employees. Schiavo-Cardoso et al. [54] showed an increase in energy and macronutrient consumption among night workers in the cleaning team of a hospital in São Paulo, when compared to day workers. In addition, a survey of airline employees reported an increase in total fat and saturated fat intake among older women on night shift [55]. Nevertheless, definitive evidence linking obesity and shift work is still lacking [50].

In the present study, the groups from São Paulo city (DSP and NSP) reported a high intake of fats. Regardless of the type of fat, consumption that falls short of or goes beyond needs is considered inadequate. Recent research indicates that the mechanism of cardiovascular disease (CVD) development may be more complex than previously thought, linking cholesterol levels to the lipid hypothesis [56]. The observed inadequacy of fat consumption in this study follows the trend observed in the *Pesquisa de Orçamentos Familiares* 2008–2009 (Household Budget Survey) [57]. This survey was carried out in all Brazilian regions and showed that a pattern of higher fat consumption accompanied the increased urbanization in different areas of the country. In addition, this hypothesis is reinforced by our finding that the TAC participants, although their fat consumption did not exceed recommendations, exhibited a significantly higher fat consumption compared to their rural counterparts (RAC). This consumption profile, associated with urbanization, might be explained by, among other factors, the progressive reduction in time dedicated to cooking food, where individuals are eating more meals outside of the home with greater use of ready-to-eat foods with a higher degree of processing [58]. This assertion may explain the results of the workers from São Paulo (DSP and NSP). However, this does not apply to the TAC group, who had a low consumption of ultra-processed foods or ready-to-eat preparations, even though these town workers had greater access to markets and cafeterias in town compared to the RAC workers. Using the 24-h food recalls could be considered a limitation because the information is based on the memory of the respondent; however, this method is judged reasonably accurate by nutritionists [28].

In the case of the TAC group, carbohydrate intake was inadequate in approximately 15% of the participants, below the standard recommended by DRIs. This group had a diet whose composition was more varied, compared to their rural counterparts, composed of vegetables, fruits, milk, meats, rice, beans, as well as cassava flour. This finding may reflect their higher purchasing power than the RAC group, allowing the purchase of, for example, more vegetables, fruit, milk, and dairy products, increasing the variety of foods and the likelihood of a balanced diet [59]. These results seem to corroborate the idea of a reduction in the consumption of basic food preparations associated with urban living mentioned earlier [58]. In Brazil, the implications of the demographic and nutritional transition process have led to changes in the pattern of food consumption, with a progressive increase in ultra-processed foods and a reduction in traditional preparations [60]. These data are confirmed by comparisons of the *Pesquisa de Orçamentos Familiares* (Household Budget Survey) 2002–2003 and 2008–2009, which show significant declines in the purchase of rice and beans, although these foods still represent a significant proportion of the diet, especially among the lowest income category [57,61].

In the present study, a significant increase in fat consumption within the urban environment, with a consequent reduction in the proportion of carbohydrates consumed was evident. This dietary change corresponds not to a reduction in simple carbohydrates but to an increase in the amount of dietary fat and a reduction in complex carbohydrates [61]. Thus, the urbanization process, besides other factors, may contribute to the differences found in the carbohydrate adequacy between the RAC and TAC groups. Carbohydrate intake was similar in the city workers of São Paulo (DSP and NSP) to levels observed in the TAC group, with the same prevalence of insufficient consumption in relation to the DRIs. However, this inadequacy was more pronounced among workers from São Paulo compared to TAC workers. By contrast, Roskoden et al. [62] observed higher carbohydrate intake among night workers when compared to day workers. According to Heath et al. [63], the greater the sleep restriction, the higher the carbohydrate consumption. These authors showed that consuming a larger amount of carbohydrate was positively associated with sleep restriction. Similar to our findings, the caloric contribution of carbohydrates was below recommended levels (45%–65%) for all groups, including day workers.

Our findings emphasize the fact that carbohydrates do not exhibit the same consumption trend that accompanies urbanization, as observed for fats. In the case of carbohydrate consumption, the reverse seems to occur, where a lower share of carbohydrates as a proportion of the total energy value of the diet is seen with higher levels of urbanization, possibly due to the increase in the fat ratio mentioned above. Insufficient protein intake was also observed in the NSP group. This result contrasts with that found by Balieiro et al. [64], who observed an increase in protein consumption among night workers when compared to day workers living in Uberlândia (Minas Gerais state – Brazil).

The lowest percentage differences observed among the study groups were related to protein consumption. In the TAC and DSP groups, protein consumption was adequate in relation to DRIs. This was not the case, however, in the RAC and NSP groups. Meat, especially beef, is the most popular source of protein in Brazil [65]. The multifactorial nature of food choices should be emphasized, with price and purchasing power being strong influencers of the process [65]. Thus, it is unsurprising that the rural workers consumed below recommended levels of proteins, considering this macronutrient has the highest cost. Protein consumption for these participants is mainly provided by local fauna. The differences in purchasing power among the study groups may be regarded as a limitation of this study. However, in general, rural workers had a lower socioeconomic status than the urban workers, indicating it would be very difficult to find populations living in different environments that have a similar purchasing power. On the other hand, the diversity of the groups studied is a strength of the study. Although the sample size may be seen as a limitation, the statistical power is high enough to allow these results to be used as a starting point to discuss future approaches for food public policies since it shows differences in food behavior according to different levels of urbanization.

Urban areas have a greater concentration of families with high socioeconomic levels and therefore are more subjected to food advertisements encouraging the consumption of ultra-processed foods, leading to overweight and obesity. The easy access to ready-to-go food increases the impact on human health, the environment, and the biosphere since it increases the number of packs, plastic, etc. [66]. The present study reinforces the need to undertake comparable population-based dietary surveys, which are uncommon in most countries [67]. Further studies should be conducted to try to minimize the nutritional transition common in urban areas.

In short, this study revealed that different levels of urbanization may have an influence on food content and timing. Moreover, the timing of eating appears to be more relevant for BMI than eating duration.

## 5. Conclusions

Our findings showed that the rural group had the earliest start time for eating. The timing of eating seems to be relevant, since an early meal start time was associated with a lower BMI among rural dwellers. Lower consumption of carbohydrate and a higher intake of fat were associated with increased urbanization. Food public policies should take into account guides regarding appropriate eating time and food intake, mainly in urban areas, which are more exposed to nutritional transition.

## Figures and Tables

**Table 1:** Sociodemographic characteristics among rural (RAC), town (TAC), and city (DSP–dayworkers; NSP–night workers) dwellers.

Variables	RAC (*n* = 22)		TAC (*n* = 19)		DSP (*n* = 11)		NSP (*n* = 13)	
	*n*	%	*n*	%	*n*	%	*n*	%
**Marital Status**								
Single	3	13.6	3	15.8	2	18.2	1	7.7
Married/living with partner	19	86.4	16	84.2	7	58.3	11	84.6
Divorced/widowed	0	0	0	0	2	18.2	1	7.7
**Children < 18 years**								
Yes	17	77.3	15	78.9	10	90.9	11	84.6
No	5	22.7	4	21.1	1	9.1	2	15.4
**Smoking**								
Yes	8	36.4	0	0	1	9.1	2	15.4
No	14	63.6	19	100	10	90.9	11	84.6
**Alcohol use**								
Yes	11	50	7	36.8	5	45.5	2	15.4
No	11	50	12	63.2	6	55	11	84.6
